# Ant-Pollinator Conflict Results in Pollinator Deterrence but no Nectar Trade-Offs

**DOI:** 10.3389/fpls.2018.01093

**Published:** 2018-08-14

**Authors:** Nora Villamil, Karina Boege, Graham N. Stone

**Affiliations:** ^1^Ashworth Laboratories, Institute of Evolutionary Biology, University of Edinburgh, Edinburgh, United Kingdom; ^2^Instituto de Ecología, Departamento de Ecología Evolutiva, Universidad Nacional Autónoma de México, Mexico City, Mexico

**Keywords:** *Turnera velutina*, ant-plant, floral nectar, extrafloral nectar, resource allocation, indirect interactions, Myrmecophily

## Abstract

Direct and indirect negative interactions between ant guards and pollinators on ant-plants are expected for two reasons. First, aggressive ants may deter pollinators directly. Second, pollinators benefit from plant investment in reproduction whilst ants benefit from plant investment in indirect defense, and resource allocation trade-offs between these functions could lead to indirect conflict. We explored the potential for ant-pollinator conflict in a Mexican myrmecophile, *Turnera velutina*, which rewards ants with extrafloral nectar and pollinators with floral nectar. We characterized the daily timing of ant and pollinator activity on the plant and used experiments to test for direct and indirect conflict between these two groups of mutualists. We tested for direct conflict by quantifying pollinator responses to flowers containing dead specimens of aggressive ant species, relative to unoccupied control flowers. We assessed indirect conflict by testing for the existence of a trade-off in sugar allocation between ant and pollinator rewards, evidenced by an increase in floral nectar secretion when extrafloral nectar secretion was prevented. Secretion of floral and extrafloral nectar, activity of ants and pollinators, and pollen deposition all overlapped in daily time and peaked within the first 2 h after flowers opened. We found evidence of direct conflict, in that presence of ants inside the flowers altered pollinator behavior and reduced visit duration, although visit frequency was unchanged. We found no evidence for indirect conflict, with no significant difference in the volume or sugar content of floral nectar between control plants and those in which extrafloral nectar secretion was prevented. The presence of ants in flowers alters pollinator behavior in ways that are likely to affect pollination dynamics, though there is no apparent trade-off between plant investment in nectar rewards for pollinators and ant guards. Further studies are required to quantify the effect of the natural abundance of ants in flowers on pollinator behavior, and any associated impacts on plant reproductive success.

## Introduction

Extrafloral nectaries, domatia, and food bodies are all means by which ant-plants (comprising myrmecophiles and myrmecophytes) (Rosumek et al., 2009; Del-Claro et al., 2016) attract and support ants by providing nesting sites or nutrients (Rico-Gray and Oliveira, 2007; Rosumek et al., 2009). In return, ants attack herbivores, prune climbing vines and prevent fungal and microbial infestation on plant tissues (Bentley, 1977; Rosumek et al., 2009). This mutualistic interaction is termed myrmecophily.

Interactions involving two or more types of mutualists of a single host are common in nature, but multispecies interactions are much less studied than pairwise and intraguild mutualisms (Strauss, 1997; Tscharntke and Hawkins, 2002; Strauss and Irwin, 2004; Adler, 2008; Melián et al., 2009; Koptur et al., 2015). To date, most research on plant-animal interactions has focused on pairwise relationships (e.g., plant-herbivore, plant-pollinator, plant-fungus) in isolation from the community in which they are embedded (Strauss, 1997; Herrera, 2000; Dáttilo et al., 2016; Del-Claro et al., 2018). This pairwise approach necessarily oversimplifies reality (Herrera, 2000) since plants interact sequentially or simultaneously with each of pollinators, herbivores, herbivore predators and pathogens (Armbruster, 1997). Furthermore, plant interactions with one partner or guild can also affect relationships with other groups or guilds (Armbruster, 1997) and alter outcomes from mutualistic to antagonistic (Strauss, 1997; Strauss et al., 1999; Herrera, 2000; Strauss and Irwin, 2004; Del-Claro et al., 2016). As a result, a growing number of studies are focusing on multispecies and multitrophic interactions (Melián et al., 2009; Fontaine et al., 2011; Nahas et al., 2012; Pineda et al., 2013; Dáttilo et al., 2016). It might be expected, for example, that the presence of predatory ants can influence pollinators, with top-down effects on plant fitness. This makes ant-plants, which rely on ants for defense against herbivores and on pollinators for seed set, a model tritrophic system in which to explore the dynamics of multispecies and multitrophic interactions.

Here, we focus on disentangling ant-pollinator interactions that occur when both mutualists share a host plant. Previous work has revealed evidence of ant-pollinator conflict in such systems (Yu and Pierce, 1998; Stanton et al., 1999; Gaume et al., 2005; Ness, 2006; Palmer and Brody, 2007; Frederickson, 2009; Stanton and Palmer, 2011; Malé et al., 2012; Assunção et al., 2014; LeVan et al., 2014; Hanna et al., 2015). Ant-pollinator conflict is expected for two main reasons. Firstly, both mutualists share with their host interest in different plant functions. Pollinators benefit from plant resource allocation to reproduction (i.e., flowers, floral nectar (FN) and pollen), whilst ants benefit from allocation to growth and defense [i.e., vegetative structures bearing extrafloral nectar (EFN) or domatia] (Yu and Pierce, 1998; Frederickson, 2009; Palmer et al., 2010). This could result in a conflict mediated by plant rewards, known as indirect conflict. Floral and extrafloral nectar share sugar as a common currency, providing potential for a trade-off and also a means of quantifying investment in each. Secondly, because ant guards actively defend their host plant as a means of protecting food and/or nesting sites, they may also repel or attack pollinators (Ness, 2006; Stanton and Palmer, 2011; Chamberlain and Rudgers, 2012), and this drawback of ant guards is known as direct conflict.

Castration is an extreme example of direct ant-pollinator conflict in which guarding ants destroy or consume the reproductive meristems, floral buds or flowers of their host plant (Yu and Pierce, 1998; Stanton et al., 1999; Gaume et al., 2005; Palmer and Brody, 2007; Frederickson, 2009; Malé et al., 2012). Such castrating behavior inevitably reduces availability of flowers and hence floral rewards for pollinators. It has been suggested that the ultimate cause of castration by patrolling ants is promoting reallocation of plant resources from reproduction to growth (Yu and Pierce, 1998; Frederickson, 2009; Malé et al., 2012), and hence increases the availability of resources on which ant colonies depend. In ant species that are obligate inhabitants of ant-plants, colony size is limited by the number of domatia (Fonseca, 1993, 1999; Orivel et al., 2011), which is positively correlated with plant investment in growth. Consequently, resource allocation strategies toward these two mutualists should be approached in a linked way because plant investment toward growth may come at the cost of investment to reproduction, and vice versa. And so, plant investment in rewards for each mutualist reward may be affected, either positively via linkage, or negatively via trade-offs. Furthermore, because plants interact with both mutualists simultaneously the presence of one mutualist may increase or decrease presence of the other.

Even in those species that do not castrate their host, ants' aggressive behaviors might threaten and deter pollinators, compromising plant reproduction (Ness, 2006; Assunção et al., 2014; LeVan et al., 2014). Avoidance of such direct conflict has been suggested to explain plant architecture or behaviors that reduce spatial (Raine et al., 2002; Malé et al., 2015; Martínez-Bauer et al., 2015) or temporal overlap of ant guards and pollinators (Gaume and Mckey, 1999; Gaume et al., 2005; Nicklen and Wagner, 2006; Ohm and Miller, 2014; Malé et al., 2015), and the presence of ant-repelling compounds which exclude ants from flowers when pollen is released (Junker et al., 2007; Willmer et al., 2009; Ballantyne and Willmer, 2012).

Extrafloral nectar is a key resource mediating multispecies interactions in many plant communities, and plants bearing extrafloral nectaries comprise up to a third of species in some biomes (Dyer and Phyllis, 2002; Rudgers and Gardener, 2004; Davidson and Cook, 2008; Dyer, 2008), particularly in tropical dry forests, savannas and *cerrados* (Rico-Gray and Oliveira, 2007; Assunção et al., 2014). The importance of ant-plants as food resources for mutualists in a given plant community is enhanced if these plants also secrete FN and pollen for pollinators. Management of ant-pollinator conflict in such a way that the crucial services provided by both mutualist groups are maintained is thus likely to be part of the adaptive landscape of many plant species. Ant-plants with extrafloral and floral nectaries represent an excellent system in which to test for trade-offs in resource allocation, competition amongst mutualistic guilds, and assess whether plant strategies minimize direct and indirect conflicts between their mutualists. To our knowledge, no study has addressed both direct and indirect ant-pollinator conflict in a single ant-plant system.

Here we tested for direct and indirect ant-pollinator conflict on a Mexican endemic ant-plant, *Turnera velutina*. In particular, we assessed whether *Turnera velutina* reduces the potential for conflict through the daily timing of FN and EFN release. We also tested for potential indirect (nectar-mediated) and direct (deterrence) conflicts between ants and pollinators. We addressed the following specific questions: (i) What are the daily timings of nectar reward secretion, ant activity, and floral visitation? (ii) Does the presence of patrolling ants deter pollinators from the flowers? (iii) Do ant species vary in their deterrence for pollinators? (iv) Are *T. velutina* plants able to re-allocate extrafloral nectar resources into floral nectar resources (increasing reward availability to flower visitors)?

## Materials and methods

### Study site and system

Field experiments were conducted in coastal sand dunes at the CICOLMA Field Station in La Mancha, Veracruz, in the Gulf of Mexico (19°36′ N, 96°22′W, elevation <100 m). The climate is warm sub-humid, with a rainy season during the summer (June to September), an annual precipitation of 1,100–1,500 mm, and a mean annual temperature ranging between 24 and 26°C (Travieso-Bello and Campos, 2006). Experiments were carried out in November 2014 at four sites with high densities of *Turnera velutina* (Passifloraceae). Greenhouse experiments were conducted in a shade house at CICOLMA.

**Figure 1:**
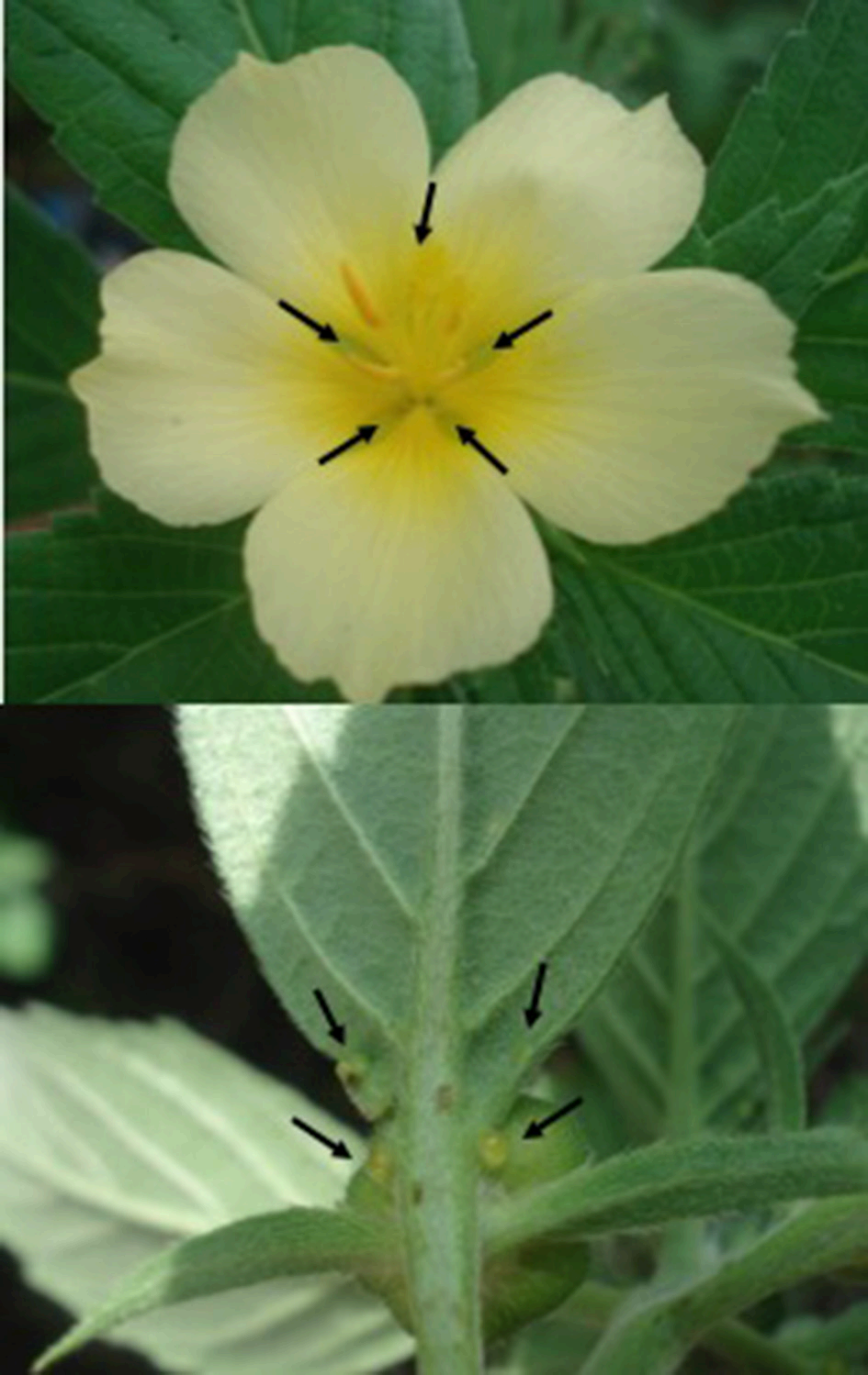
Flower of Turnera velutina **(top)** with arrows indicating the location of the floral nectaries at the base of the corolla, between the petals, and leaves **(bottom)** with arrows indicating the location of extrafloral nectaries, on the underside of the leaves.

*Turnera velutina* is an endemic perennial shrub (Arbo, 2005) and myrmecophile (Cuautle and Rico-Gray, 2003). At La Mancha, *T. velutina* is patrolled by at least seven ant species (Cuautle et al., 2005; Zedillo-Avelleyra, 2017) and its main herbivores are the specialist caterpillars of the butterfly *Euptoieta hegesia* (Nymphalidae). Extrafloral nectar is provided in paired cup-shaped glands located at the bottom of the leaf blade at the junction with the leaf petiole, on the underside of the leaves (Figure 1A, Elias et al., 1975; Villamil et al., 2013). Although it flowers year-round, flowering peaks during summer (Cuautle et al., 2005). Flowers last one day, are animal-pollinated (Sosenski et al., 2016) and provide FN at the base of the corolla (Figure 1). Honeybees (*Apis mellifera*) are the dominant pollinators at La Mancha, accounting for 94% of the visits (Sosenski et al., 2016) and collect both pollen and floral nectar, but the role of other floral visitors is yet to be investigated as effective pollinators. There is no spatial segregation of patrolling ants and floral visitors in *Turnera velutina* since flower buds emerge from the axillary meristems of leaves bearing extrafloral nectaries (Villamil, 2017). Furthermore, EFN volume and sugar content are higher at the flower stage than for either buds or fruits (Villamil, 2017).

### Fieldwork methods

#### Mutualist activity curves: patrolling ants and pollinators

We quantified daily activity of patrolling ants and flower visitors on *T. velutina* by surveying flowers (*n* = 120 plants, *n* = 1,604 flowers) and their associated leaves at all four La Mancha sites in November 2014. We observed all open flowers for 2 min every hour throughout anthesis (0800–1300 h), with one observer at each site. Every hour, we counted the total number of floral visitors and ants patrolling extrafloral nectaries across all flowers within a site. We sampled the same sites over multiple days. Since these are one-day flowers, we considered each site-day as a replicate (*n* = 10 site-days; 43.23 ± 2.89 flowers/site-day), and incorporated site-and-day effects into our statistical modeling (see below).

#### Nectar secretion and pollen deposition curve

Nectar secretion and pollen deposition data were collected from 4 sites within CICOLMA over 5 consecutive days in September 2015. We visited a single site per day (with one exception which was visited twice) and sampled FN and EFN secretion rate and pollen deposition from 1 flower per plant for 5 plants per site (*n* = 20 plants). Flowers were sampled every hour during the anthesis period (0800–1300 h). Flowers were bagged with tulle bags before anthesis and FN and EFN were collected every hour during anthesis. The first collection was taken as soon as the corolla was fully open. Flowers were re-bagged between measurements to avoid nectar consumption and a masking tape band with Tanglefoot was applied on the stem below the flower to exclude ants for the duration of the experiment. FN was extracted using a 1 μl capillary inserted into each of the five nectaries in a single flower (Minicaps Disposable capillaries, Hirschmann Laborgerate, In 20°C, ISO 7550, R <0.5%, CV <1.0%, Germany). EFN was also collected from the glands using 1 μl capillaries. Nectar volume was measured using a digital caliper (Mitutoyo Digimatic) and sugar concentration was determined in °Brix (g sucrose per 100 g solution) using a 0–50°B hand held refractometer (Reichert, Munich, Germany). To obtain all of the sugar from extrafloral nectaries, the glands were washed with 2 μl of deionized water using a 0–5 μl micropipette and the sugar concentration of the wash was quantified using the refractometer. Total sugar content in FN and EFN was calculated from volume and °Brix values according to Comba et al. (1999) using the formula:

$$\begin{array}{l} s=dvC/100 \end{array}$$

where *s* is the sugar content (μg), *v* is the nectar or wash volume (μl), and *d* is the density of a sucrose solution at a concentration *C* (g of sucrose per 100 g solution) as read on the refractometer. The density was obtained according to Comba et al. (1999) using the formula:

$$\begin{array}{l} d=0.0000178C^{2}+0.003791C+0.9988603 \end{array}$$

A different flower from each of these twenty plants was chosen and marked, but never bagged. We collected one stigma every hour, to sample the pollen deposited 1 h, 2 h, and 3 h post-anthesis. A small proportion of flowers contained a fourth stigma, contributing to a small dataset for 4 h post anthesis. Stigmas were stored individually in labeled Eppendorf tubes. Stigmas were individually mounted on slides for fresh squash glycerin preparations. Slides were sealed using nail polish and kept in a fresh and dry environment at 22°C. Each slide was labeled with the site, day, hour, and plant identity. The number of pollen grains on each stigma was counted under a microscope, and changes in numbers at each time interval plotted to generate the pollen deposition curve.

#### Direct conflict

To test whether non-ant flower visitors detect and avoid flowers with ants, visitation was observed in flowers with and without dead ants for 4 flowers on each of 40 plants (*n* = 160 flowers). Plants > 80 cm in height were haphazardly selected and flowers bagged at 0700 before anthesis. Once the corollas had opened, three ant corpses from a single ant species (either *Dorymyrmex bicolor, Brachymyrmex* sp., or *Paratrechina longicornis*) were placed inside each of three flowers/plant. A fourth flower was left as an ant-free control. Ant corpses were placed on the inner surface of the petals in each flower. These species were chosen because they were amongst the most abundant species (see Results), displayed patrolling behaviors on *T. velutina*, and were detected as potentially differing in their effects on *Apis mellifera* pollinators by a previous study (Villamil et al. unpublished data). Additional flowers were removed from the plant to standarise floral display across all individuals. Ants were killed in 50% ethanol, which was allowed to evaporate for 30 min at ambient temperature (28–35°C) from all samples to prevent ethanol vapors from influencing pollinator behavior. Flowers containing dead ants were observed for 20 min, recording pollinator identity, visit frequency and duration, and associated pollinator behaviors. Observations were conducted during October-November 2016 with four simultaneous observers collecting data from different plants.

When assessing the effects of ants inside the flowers on pollinator visitation we only considered visits by *Apis mellifera*, since this is the dominant *T. velutina* pollinator in this population (Sosenski et al., 2016) and accounted for 91% of the visits in this experiment. We classified honeybee behaviors as “inspection” or “contact.” Inspection behaviors comprised either approaching or hovering over a particular flower without landing. Contact behaviors were those that occurred inside the flower, between landing and take-off, and comprised foraging on pollen or nectar resources or standing on the petals, anthers or stigmas.

#### Indirect conflict

To test possible trade-offs in plant resources between FN and EFN we conducted a greenhouse experiment on 72 plants from 18 different maternal lines (2–4 siblings per maternal family, generated from field individuals). Plants were kept under greenhouse conditions in a shade house located within CICOLMA field station. Plants were grown in 1L plastic pots with a substrate of local soil and vermiculite (50:50) and watered every other day (for rearing details see: Ochoa-López et al., 2015). During the experiment, plants were watered every night, and extrafloral nectaries were sprayed with water to wash away any nectar secretion from previous days and to prevent fungal infections on the glands. Pre-anthesis buds were bagged either the night before or during the morning before anthesis to prevent any nectar theft by unexpected insects that may occasionally enter the shade house.

Pairs of maternal siblings were chosen and randomly assigned to either control or experimental groups. The extrafloral nectaries in all leaves of experimental plants were clogged by applying Mylin transparent textile paint in the nectary cup. Extrafloral nectaries from control plants were left intact (unclogged) and droplets of the same paint were applied on the leaf blade above the glands to match any unintended effects on plants across treatments. Very young floral buds were marked, and their extrafloral nectaries clogged from their emergence, throughout their development, and until anthesis. The droplets on the extrafloral nectaries or leaf blades were checked daily and replenished when required, especially when a new leaf emerged in order to guarantee uniform and continuous application of the clogging treatment across all leaves.

FN secretion was measured before the clogging treatment was applied, and once again after the young clogged/marked buds became flowers. The aim was to test whether flowers that were unable to secrete EFN invested more sugar resources in floral nectar. FN was collected from control and treatment flowers between 1300 and 1500 h using a 1 μl microcapillary pipette. Nectar volume and total sugar mass was estimated and calculated as described above.

### Statistical methods

All statistical analyses were conducted in R version 3.23 (R Core Team 2016). All mixed effects models were fitted using the ‘multcomp’ R package (Bates et al., 2016) and *post hoc* Tukey comparisons were fitted using the ‘multcomp’ R package (Hothorn et al., 2008), unless stated otherwise.

#### Mutualist activity and reward secretion curves

To test whether ant or pollinator activity changed over daily time, we fitted a Poisson mixed model with either the number of ants patrolling or the number of floral visitors as the response variable. We fitted time of day as a fixed effect, with linear and quadratic terms to detect non-linear activity patterns over time. The number of flowers per site was fitted as a log-transformed offset to control for floral display, since we recorded visitor counts per site rather than counts per individual flower (see fieldwork methods). Flowers of *T. velutina* last for a single day, and because multiple flowers were sampled on a given site on a given day, we fitted site identity as a random effect to account for differences between site and day variation in variables that could influence ant abundance, such as resource availability, ant diversity, or the abundance/proximity of ant nests. We also included an observation-level random effect (OLRE) where each data point receives a unique level of a random effect to control for overdispersion (Hinde, 1982).

To test if FN and EFN secretion changed over the anthesis period we fitted a Poisson mixed model independently for each nectar type, with sugar mass (μg) as the response variable. Nectar sugar content is usually estimated in μg, and we report our raw data in such units. However, to facilitate model convergence we re-scaled our response variable (sugar content) from μg to g, and rounded it to the next integer to better fit a Poisson distribution. We fitted time of day as a fixed effect, with linear and quadratic terms to detect non-linear activity patterns over time. We fitted plant identity as a random effect, and, included an observation-level random effect.

#### Timing of daily activity and secretion peaks

We computed the time at which mutualist activity and nectar secretion reached their maximum by calculating the time at which the slope (i.e., the differential of the fitted model with respect to time) for each variable is zero and then solving for hour, as follows:

$$\begin{array}{l} y=\;\beta_{hour}*hour+\;\beta_{hour^{2}}*hour^{2}\\ \;\frac{dy}{d_{hour}}=\;\beta_{hour}+2\beta_{hour^{2}}*hour\\ \;0\;=\;\beta_{hour}+2\beta_{hour^{2}}*hour\\ \;\frac{-\;\beta_{hour}}{2\beta_{hour^{2}}}=\;hour\\ \;hour=\;-\frac{1}{2}*\frac{\beta_{hour}}{\beta_{hour^{2}}} \end{array}$$

#### Direct conflict

The effect of different ant species on the visitation frequency was tested using Poisson mixed effects models. We fitted the number of visitors as the response variable, and ant species inside the flower (Control, *Dorymyrmex bicolor, Brachymyrmex* sp., or *Paratrechina longicornis*) was fitted as a fixed effect. Because these are 1 day flowers, plant-day identity was chosen as a random effect to control for individual variation in floral and extrafloral nectar investment. We also included an observation-level random effect. *Post hoc* Tukey comparisons were used to test differences in visit duration between the four treatments.

We tested whether ant species inside the flower differed in their effect on the likelihood with which a pollinator displayed an inspection behavior using a binomial mixed model. The presence or absence of inspection behaviors was coded as the response variable and ant species was fitted as a fixed effect. As random effects we fitted the plant-day identity, and the visitor identity. For those visits where the pollinator displayed an inspection behavior, we fitted the proportion of time per visit spent displaying inspection behaviors using a Gaussian mixed model with logit transformation for data normality. Ant species was included as a fixed effect, and plant-day identity, and the visitor identity were fitted as random effects.

Finally, differences in the duration of inspection or contact behaviors in flowers containing different ant species were analyzed using Gamma mixed models. Mixed effects models were fitted independently for each behavior, but using the same model structure fitting visit duration per flower as the response variable. Ant species inside the flower was fitted as a fixed effect. Plant-day identity was chosen as a random effect to control for individual variation in floral and extrafloral investment, and daily weather variations. We also included an observation-level random effect. *Post hoc* Tukey comparisons were used to test differences in visit duration between the four treatments.

#### Indirect conflict

For each plant, we estimated the difference in FN produced before and after the extrafloral nectaries were clogged as follows: *D*_*FN*_ = *Post*_*FN*_ − *Pre*_*FN*_, where *D*-_FN_ is the difference, *Post*_FN_ is the FN production after the extrafloral nectaries secretion was prevented and *Pre*_FN_ is the FN production before the extrafloral nectaries were clogged. Differences in volume and sugar content between control and experimental flowers were tested using mixed effects models. Both variables had normal distributions and so Gaussian mixed effects models were fitted using the same model structure: The clogging treatment was fitted as a fixed effect, and the maternal family was fitted as a random effect to independently explain variation in both nectar volume and sugar content.

## Results

### Mutualist activity, reward secretion, and pollen deposition curves

**Figure 2:**
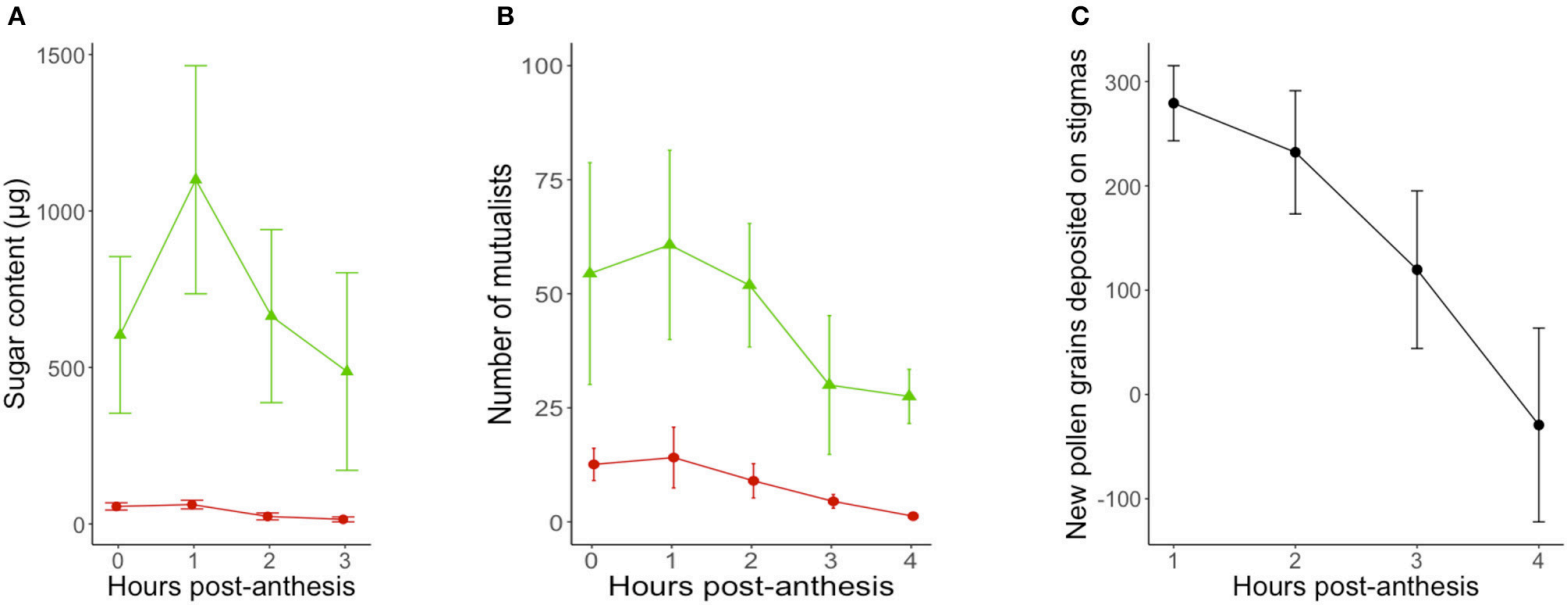
Timing of **(A)** reward secretion (*n* = 20 flowers-leaves), **(B)** mutualist activity (n = 1,604 flowers; *n* = 10 sites/day) and **(C)** pollen deposition (*n* = 20 flowers) in *Turnera velutina* during the anthesis period (08:30–12:30), showing raw data from field observations (mean ± se). Red circles show floral nectar and the activity of pollinators in flowers, whilst green triangles show extrafloral nectar and the activity of ants at extrafloral nectaries.

Activity curves show that both patrolling ants and floral visitors were most active within the first 2 h post-anthesis (Figure 2), although the visitation peak by potential pollinators predicted from model estimates was on average over an hour before the predicted peak for ant activity (9 min post-anthesis for potential pollinators, 90 min post-anthesis for ant patrolling; Table 1).

**Table 1:** Model statistics for the timing of mutualists activity, reward secretion and pollen deposition in *Turnera velutina*.

	**Response**	**Fixed effects**	**N**	**Estimate**	**LRT**	***P*****-value**		**Random effects**	**Variance**	**SD**	**Maxima estimates**		
											**Peak hour**	**mpa**	**Time of day**
Mutualist activity	Patrolling ants (number)	Log(flowers)	33	0.804	7.967	0.004	^***^	OLRE Site ID	0.221 0.462	0.471 0.680	1.5	90	09:30
		Hour	33	0.088	0.148	0.699							
		Hour^2^	33	−0.028	0.201	0.653							
	Floral visitors (number)	Log(flowers)	42	0.507	2.619	0.105		OLRE Site ID	0.097 0.991	0.311 0.991	0.15	9	08:09
		Hour		0.040	0.036	0.849							
		Hour^2^		−0.131	3.838	0.05	^*^						
Reward secretion	Floral sugar (μg)	Hour	78	0.1445	0.1019	0.7495		OLRE Plant ID	0 0	0 0	0.39	23	08:23
		Hour^2^	78	−0.1818	1.3222	0.2502							
	Extrafloral sugar (μg)	Hour	78	0.4647	1.0070	03156		OLRE Plant ID	0.2425 0.4013	0.4925 0.6335	1.13	68	09:08
		Hour^2^	78	−0.2053	1.8949	0.1686							
	Pollen deposition (number of grains)	Hour	44	0.3240	21.38	3.73^−06^	^***^	OLRE Site ID	0.1049 0.0957	0.3240 0.3094			

*Estimates at which the maxima for mutualist activities and reward production are reached are shown in the last three columns. Peak hour is the time in hours estimated, mpa shows minutes post-anthesis when the maxima is reached, and time of day indicates an approximation of when that activity is likely to occur, although this varies depending on the season and the time of sunrise*.

* *P < 0.05*;

*** *P < 0.001*.

On average, a flower and its associated leaf secreted a total of 2,815 ± 767 μg of sugar via floral and extrafloral nectar throughout the 4.5 h anthesis period. The total sugar content in FN was 149 ± 19.3 μg of sugar, whilst total extrafloral sugar was 2,665 ± 765 μg. Thus, the relative sugar contributions of floral and extrafloral nectar in a leaf-flower module were 5.3 and 94.7%, respectively.

Floral and extrafloral nectaries of *T. velutina* are both able to quickly replenish nectar after experimental removal by non-destructive sampling of the same flowers over the entire anthesis period (Figure 2A). FN and EFN are secreted simultaneously during anthesis and with the highest amount of sugar content secreted during the first 2 h of anthesis (Figure 2A), although their secretion peaks predicted from model estimates are slightly mismatched (Table 1). EFN secretion peaked 68 min after our first collection, which was taken as soon as flowers were fully open, whilst FN peaked 23 min after the first collection. The timing of peaks in secretion of the two types of nectar matches that for the mutualists that harvest each resource. Peak pollen deposition occurred at the beginning of anthesis and steadily declined over time (Figure 2C). Hence, pollen deposition data were analyzed using a linear model without fitting hour as a squared term and we did not estimate timing of daily maxima using model derivations (Table 1).

We recorded 1,535 ant visitors from nine ant species patrolling extrafloral nectaries of *T. velutina* at CICOLMA (Table 2). *Dorymyrmex bicolor, Paratrechina longicornis*, and *Brachymyrmex* spp. accounted for 68.5% of the total ants observed, and 77.35% of the patrolling ants, after excluding *Monomorium* spp. that were never observed displaying patrolling behaviors on *T. velutina* and are mostly parasitic consumers of FN and EFN (lestobiotic) (Ettershank, 1966; Bolton, 1987).

**Table 2:** Abundance and identity of the ants recorded patrolling extrafloral nectaries during the 2014 census and the floral visitors recorded during the direct conflict experiment in 2016 on Turnera velutina plants.

	**Taxon**	**Visitors**	**Percentage**	**Subfamily**	**Patrolling**
Ants at extrafloral nectaries	*Paratrechina longicornis*	487	31.72	Formicinae	Gregarious
	*Dorymyrmex bicolor*	421	27.42	Dolichoderinae	Gregarious
	*Monomorium ebenium*	270	17.58	Myrmicinae	Gregarious
	*Camponotus planatus*	184	11.98	Formicinae	Loner
	*Brachymyrmex* sp.	73	4.75	Formicinae	Gregarious
	*Camponotus mucronatus*	60	3.90	Formicinae	Loner
	*Crematogaster* sp.	23	1.49	Myrmicinae	Gregarious
	*Camponotus novogranadensis*	15	0.97	Formicinae	Loner
	*Pseudomyrmex gracilis*	2	0.13	Pseudomyrmicinae	Loner and very rare
Floral visitors	*Apis mellifera*	907	91.5		
	Native bees	61	6.05		
	Diptera	11	1.10		
	Lepidoptera	10	1		
	Coleoptera	1	0.1		
	Wasps	1	0.1		

### Direct conflict

We recorded 991 floral visitors, of which 907 (91.5%) were by the honeybee *Apis mellifera*. Of the remaining 8.5%, 61 visits were by native bees, 11 by flies (Diptera), 10 visits by Lepidoptera, one visit by a beetle (Coleoptera) and one by a wasp (Hymenoptera) (Table 2).

**Figure 3:**
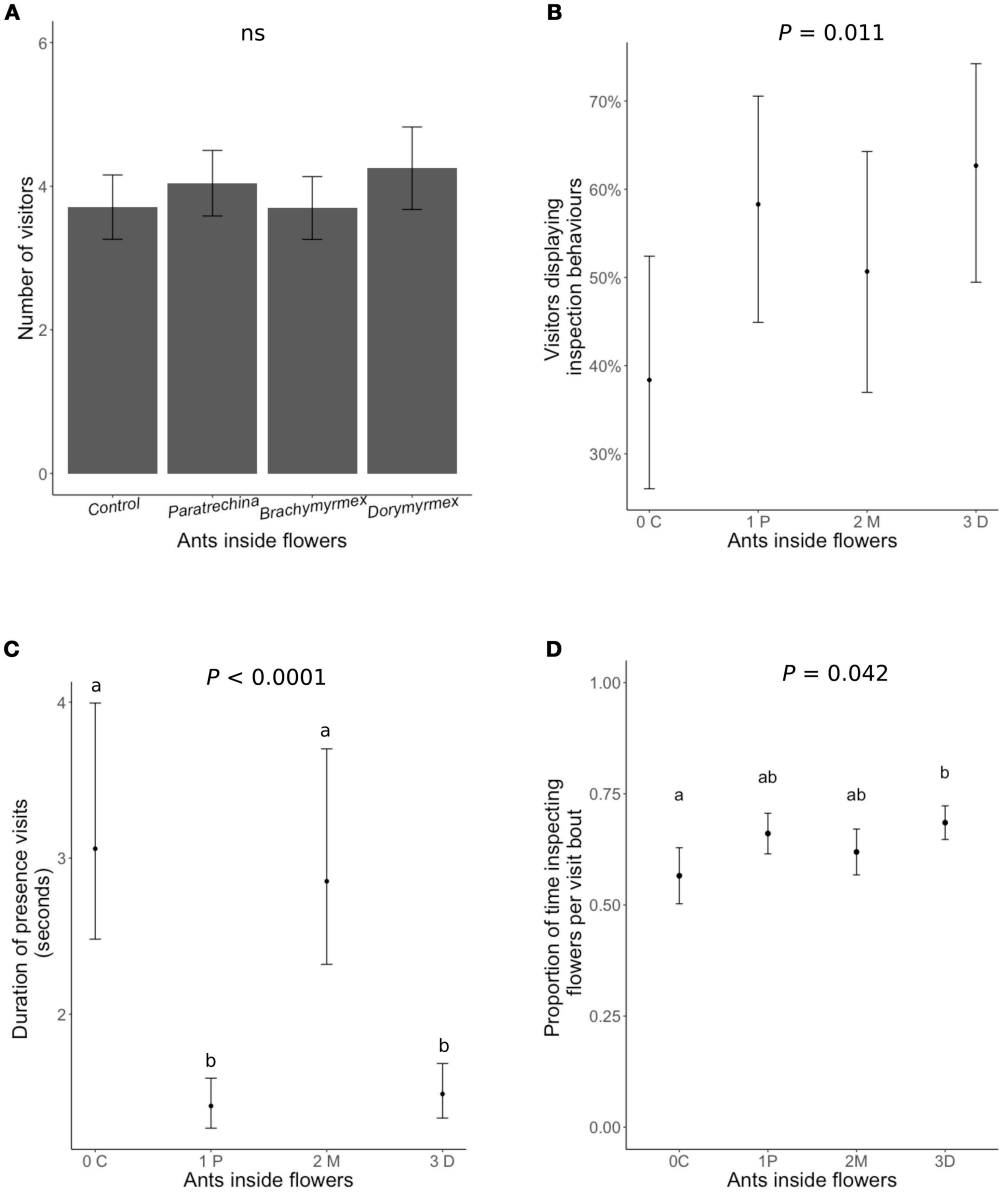
The effect of dead ants inside the flowers of *Turnera velutina* on different aspects of the behavior of visiting honeybees: **(A)** visitation frequency, **(B)** the percentage of visitors of displaying inspection behaviors, **(C)** the duration of contact visits (time spent inside the flower), and **(D)** proportion of time spent inspecting the flowers per visit bout (hovering over the floral head space). Ant species are arranged in order of increasing aggressivity and names are abbreviated: C, control with no ants; 1P, *Paratrechina longicornis*; 2M, *Brachymyrmex* sp.; 3D, *Dorymyrmex bicolor* ants. (*n* = 40 plants; *n* = 160 flowers).

Neither the presence of ants inside flowers nor their identity had any significant effect on the number of honeybees visiting the flowers (Figure 3A, Table 3). The presence of the most aggressive ant species, *Dorymyrmex bicolor*, increased the likelihood of a pollinator displaying inspection behaviors by 20% (Figure 3B), and increased by 12% the proportion of time per visit spent displaying inspection behaviors rather than foraging or pollinating the flower (Figure 3C). Finally, the presence of *Dorymyrmex bicolor* and *Paratrechina longicornis* inside the flowers halved the duration of contact visits compared to control flowers without ants, or to flowers with *Brachymyrmex* sp. ants inside.

**Table 3:** Model statistics for the experiments testing indirect (nectar-mediated) and direct (pollinator deterrence) ant-pollinator conflict.

	**Response**	**Distribution**	**Fixed effects**	**N**	**LRT**	***P*****-value**		**Random effects**	**Variance**	**SD**
Indirect conflict	Floral nectar (μl)	Gaussian	Clogging treatment	50	0.0813	0.7754		Family	0.6987	0.8359
	Floral nectar (μl)	Gaussian	Clogging treatment	50	0.21006	0.6467		Family	138.5	11.77
Direct conflict	Number of visitors	Poisson	Ants in flowers	95	1.1848	0.7567		OLRE Plant	2.77^e−08^ 0.144	0.0001 0.3803
	Likelihood of being alerted	Binomial	Ants in flowers	373	10.715	0.01337	^*^	ID visitor Plant	8.279^e−10^ 0.595	2.877^e−05^ 0.7715
	Proportion of time per visit spent displaying inspection behaviors	Gaussian (logit)	Ants in flowers	112	7.5728	0.055	^*^	ID visitor Plant	0.0009313 0.0201	0.03052 0.14198
	Duration of presence behaviors (sec)	Gamma	Ants in flowers	307	392.37	2.2^*e*−16^	^***^	OLRE ID visitor Plant	0.1202 0.001587 1.928^e−10^	0.3468 0.03984 0.001389

* *P < 0.05*;

*** *P < 0.001*.

### Indirect conflict

**Figure 4:**
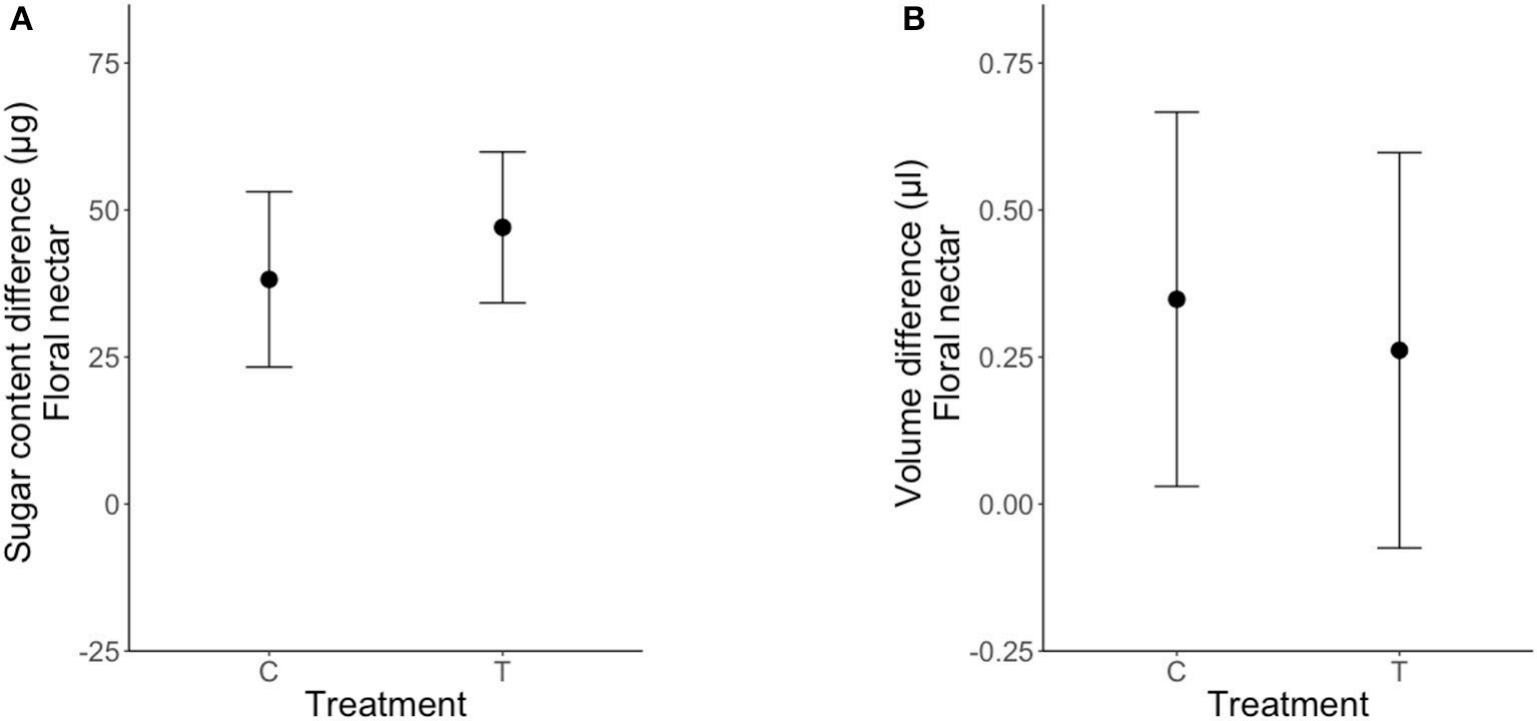
Effect of clogging on nectar secretion and sugar re-allocation in *Turnera velutina* for nectar volume **(A)** and sugar content **(B)**, between control (C) and treatment (T) plants (means ± se; *n* = 50 plants). Values shown are a difference in floral nectar, defined as: [FN post-treatment–FN pre-treatment].

Clogging extrafloral nectaries on the leaves associated with newly emerged floral buds had no effect on their FN volume [LRT _(1, 49)_ = 0.21; *P* = 0.64, Table 3, Figure 4B] or sugar content [LRT _(1, 49)_ = 0.087; *P* = 0.77, Table 3, Figure 4A]. Differences in FN volume and sugar content (FN_post−treatment_ − *FN*_pre−treatment_) were positive in plants under both control and clogged treatments (Figure 4).

## Discussion

Our findings show that both ants and pollinators are active while flowers are open (Figure 2B), that FN and EFN are simultaneously secreted (Figure 2A), and that pollen deposition occurs when ants are actively patrolling (Figure 2C). Consequently, in *T. velutina* guarding ants and pollinators operate in close spatial and temporal proximity, implying that direct and indirect conflict could occur in this system. We found, however, no evidence for indirect, nectar-mediated, conflict between ants and pollinators, since plants did not reallocate resources toward floral nectar, even when 95% of the sugar investment during anthesis, which is in EFN, is prevented (Figure 2). We found evidence for direct conflict, as the presence of dead individuals of patrolling ant species inside flowers was associated with both higher frequency of inspection behaviors in potential pollinators, and reduced visit duration (time spent inside flowers) (Figure 3). Taken together, these effects increased handling time per flower and reduced pollinator foraging efficiency. Nonetheless, this result was obtained under an experimental setting using dead ants placed inside flowers. Further studies are required to test (i) the effect of living patrolling ants on pollinator visitation, and (ii) the impact of any such effects on plant fitness. The latter are crucial to understanding whether there is ongoing selection on *Turnera velutina* to manage direct ant-pollinator conflict.

### Mutualist activity, reward secretion and pollen deposition

Floral and extrafloral nectar were secreted simultaneously and rapidly replenished in *T. velutina*, especially during the first 2 h post anthesis (Figure 2). Replenishment is a general feature of EFN secretion (Pacini et al., 2003; Pacini and Nepi, 2007; Pacini and Nicolson, 2007). In fact, we are unaware of any report documenting extrafloral nectaries incapable of replenishing secretion after consumption (Pacini et al., 2003; Pacini and Nepi, 2007; Pacini and Nicolson, 2007; Escalante-Pérez and Heil, 2012; Orona-Tamayo et al., 2013; Heil, 2015). In contrast, species vary in whether FN is replenished or not (for details on floral nectar dynamics see: Pacini et al., 2003; Nicolson et al., 2007; Willmer, 2011). We suggest that rapid resupply is crucial in short-lived flowers, such as the one-day flowers of *Turnera* species, because it makes the flower attractive again for another visit, potentially increasing pollen transfer, pollen deposition, and seed set. Interestingly, Dutton et al. (2016) reported no FN resupply in flowers from three congeners (*Turnera ulmifolia, Turnera subulata*, and *Turnera joelii*), when sampling FN in the morning and afternoon, finding no FN secretion in the afternoon collection. These observations show either variation in nectar resupply within closely related species displaying similar floral biology, or perhaps suggest that shorter term dynamics in the nectar supply of the other three species were not detected by the sampling methods used. The latter highlights the need to test floral resupply within short time scales, because over long sampling intervals, flowers can both secrete and reabsorb nectar (Pacini et al., 2003; Nicolson et al., 2007; Willmer, 2011). Finding no standing crop when a flower is resampled in the afternoon does not rule out replenishment after emptying in the morning, and then reabsorption later in the day (Kearns and Inouye, 1993).

Model-based estimation of the daily timing of the maxima of nectar secretion suggest that floral nectar secretion peaks a few minutes after the corolla is fully open (Table 3) and 45 min before peak EFN sugar secretion (Figure 2A). This represents a slight mismatch in the timing of rewards for ants and pollinators, which may underlie the 85 min mismatch in estimated peaks of ants and floral visitor activity (Table 1). To our knowledge, temporal segregation in the activity of ants and pollinators has been reported only for obligate (myrmecophytic) species patrolled or tended by a single ant species at a time (*Humboltia brunonis* (Fabaceae; Gaume et al., 2005), *Hirtella physophora* (Chrysobalanaceae; Malé et al., 2015), and *Opuntia imbricata* (Cactaceae; 2014)). In some specialized systems, ant and pollinator activity occurs in close proximity and simultaneously, but conflict is prevented by ant-repellent floral volatiles [*Vachellia zanzibarica* (Fabaceae; Willmer and Stone, 1997)] and *Vachellia hindsii* (Fabaceae; Raine et al., 2002). On the other hand, temporal overlap in ant activity at extrafloral nectaries and pollinator visitation to flowers has been reported for facultative ant-plants associated with many ant species simultaneously [*Vachellia constricta* (Fabaceae; Nicklen and Wagner, 2006), *Acacia myrtifolia*, (*Acacia sensu stricto*, Fabaceae; Martínez-Bauer et al., 2015)*, and Heteropterys pteropetala* (Malpighiaceae; Assunção et al., 2014)]. Our results add *Turnera velutina* to the list of facultative myrmecophiles with synchronized ant and pollinator activity (Figure 2). This synchronous myrmecophile vs. segregated myrmecophyte pattern is consistent with evidence that ants in obligate mutualisms are more aggressive and better defenders (Chamberlain and Holland, 2009; Rosumek et al., 2009; Trager et al., 2010), but may impose greater ecological costs on host pollination. We suggest that temporal segregation of mutualists and/or ant repellent floral volatiles are alternative strategies that reduce such costs. Further studies on the timing of pollinator, ant visitation and ant aggressivity in a wider range of systems are required to test the temporal component of this hypothesis.

### Direct conflict

We showed that dead ants inside the flowers of *T. velutina* have an impact on honeybee behavior (Figure 3). Ant presence was correlated with shorter honeybee flower visits (Figure 3C), an increase in the proportion of visitors displaying inspection behaviors, and increased duration of inspection behaviors per visiting bout (Figure 3). We interpret longer inspection behavior to indicate increased caution in the bees (as previously assumed by: Altshuler, 1999; Ness, 2006; Junker et al., 2007; Assunção et al., 2014; Cembrowski et al., 2014). Our findings are consistent with work on *Heteropterys pteropetala* in which plastic ants inside flowers negatively affected pollination (Assunção et al., 2014). Results for *H. pteropetala* differ from ours in that the bees pollinating *H. pteropetala* showed significantly reduced visitation rates to flowers containing plastic ants. In contrast, honeybees in *T. velutina* did not visit flowers containing ant corpses less frequently than control flowers (Figure 3A). In both systems, ants feeding at extrafloral nectaries did not hinder pollination (Assunção et al., 2014; Villamil et al., unpublished data). This suggests that while pollinators avoid ants in flowers, plants may have evolved other mechanisms to prevent ants from entering flowers, resulting in only rare encounters between ants and pollinators.

Although experiments that place ant cues on flowers can tell us about the response of pollinators to ants, they must be interpreted with caution as an indicator of current ant-pollinator conflict. Firstly, because ants may rarely enter flowers (Villamil et al. submitted). Secondly, by placing such ant cues in flowers we may be violating existing ant-excluding or ant-repelling plant mechanisms (Willmer, 2011). Thirdly, in contrast to such experimental treatments, ants do not naturally remain in the flowers for long periods (Assunção et al., 2014), and only a low proportion of flowers may be occupied at any one time. For instance, in *T. velutina* only 10% of the flowers are occupied by ants (Villamil et al., submitted).

### Does herbivore deterrence match pollinator deterrence?

Although some studies have documented variation among ant species in aggression toward herbivores (Ness, 2006; Miller, 2007; Ohm and Miller, 2014), little is known about the effect of different patrolling ant species with varying levels of aggressivity on pollinator visitation (Ness, 2006; Miller, 2007; LeVan et al., 2014; Ohm and Miller, 2014). Nonetheless, a positive correlation between the level of defense provided and the level of pollinator deterrence they exert has often been assumed since ant traits involved in defense (patrolling activity and aggressivity) are likely to be the same as those involved in pollinator deterrence (Ohm and Miller, 2014). Bees tend to forage in a way that maximizes the net benefit of each foraging trip (Stephens and Krebs, 1986; Jones, 2010; Cembrowski et al., 2014). When foraging in ant-plants, this benefit might be maximized if foragers avoid flowers or patches where predation risk is high (Dukas, 2001; Dukas and Morse, 2003; Ness, 2006; Jones and Dornhaus, 2011; Assunção et al., 2014), as could be the case when encountering ant species that attack pollinators. Some ants also consume FN and pollen, and such plants may represent high risk foraging environments with low net rewards for pollinators (Ness, 2006). Shorter or fewer visits to such flowers may be a pollinator strategy to maximize foraging efficiency by avoiding flowers, plants, or patches with high predation risk (Jones and Dornhaus, 2011).

In *T. velutina*, the most aggressive ant guard, *Dorymyrmex bicolor*, had the strongest effect on pollinator behavior (Figure 3), while *Brachymyrmex* sp. ants inside the flowers did not reduce the duration of pollinator visits. The least effective anti-herbivore ant species, *Paratrechina longicornis* (Villamil unpublished data), halved the duration of pollinator visits (Figure 3). In *Ferocactus wislizeni*, plants tended by *Solenopsis xyloni*, the most aggressive ant species, had fewer and shorter pollinator visits (Ness, 2006). Such differences are consistent with pollinator sensitivity to ant aggressiveness. In contrast, although ant exclusion in *Opuntia imbricata* significantly increased pollinator visitation, there were no differences in impacts associated with different ant species (Ohm and Miller, 2014), and no evidence that the more aggressive guard (*Liometopum apiculatum*) had a stronger deterring effect on pollinators (Ohm and Miller, 2014). Whether the level of ant aggressivity toward herbivores correlates positively with the ecological costs on pollination via pollinator deterrence remains unknown (but see: Ness, 2006; Miller, 2007; LeVan et al., 2014; Ohm and Miller, 2014), and should be tested, not assumed.

### Indirect conflict

Our experimental approach found no evidence for a trade-off in sugar investment in extrafloral and floral nectar in *T. velutina*. We conclude that there is no indirect nectar-mediated conflict between guarding ants and pollinators in *Turnera velutina*, and that pollinators do not obtain greater rewards when rewards for patrolling ants are eliminated.

We found only two previous studies testing indirect, nectar-mediated ant-pollinator conflict by quantifying sugary rewards (FN and EFN) to both mutualists (Chamberlain and Rudgers, 2012; Dutton et al., 2016). Previous work on other *Turnera* species by Dutton et al. (2016) found evidence of a trade-off in two of the three *Turnera* species tested; removing EFN decreased FN and *vice versa* in *T. ulmifolia* and *T. subulata*, but not in *T. joelii*. Interestingly, both *Turnera* species in which trade-offs were detected by Dutton et al. (2016) invested equally in FN and EFN, whilst *T. joelli* (which showed no trade-off) invested more in EFN (Dutton et al., 2016). The same pattern holds for *T. velutina*, a species with an asymmetric investment toward EFN, which accounts for 95% of the sugar allocation per leaf-flower module, and where we found no trade-off or resource reallocation from EFN to FN (Figure 4). Unfortunately data on FN and EFN volume and sugar content were not reported for the cotton species (Chamberlain and Rudgers, 2012).

One possible reason for lack of a trade-off is that sugar is not a limiting resource for the plant. If so, there would be no reason to expect dynamic reallocation. Estimates of the metabolic costs of nectar secretion vary, and while some studies suggest low metabolic costs (O'Dowd, 1979: EFN accounts for 1% of the total energy invested per leaf), others indicate investment of up to 37% of daily photosynthesis in floral nectar (Southwick, 1984; Pyke, 1991). A second reason, which applies in particular to comparative cross-species analyses rather than experimental manipulations, is that investment in both forms of nectar may be influenced by other aspects of life history strategy. Chamberlain and Rudgers (2012) found no significant negative correlations between extrafloral nectary and floral traits in a comparative analysis across cotton (*Gossypium*) species, and correlations were significantly positive in 11 of 37 cotton species. Foliar extrafloral nectary volume was positively associated with plant investment in floral nectar, rejecting the hypothesis of trade-offs among investments in pollinators versus bodyguards in *Gossypium*. Several potential mechanisms underlying the positive correlations between FN and EFN have been proposed, including pleiotropy, and genetic, physiological or ecological linkage (Chamberlain and Rudgers, 2012). The pleiotropy or genetic linkage hypothesis could be tested using genome sequencing (Chamberlain and Rudgers, 2012). Positive correlations could also arise from physiological or ecological linkage. Traits such as FN and EFN may be physiologically linked. However, the fact that in *Gossypium* FN volume was most strongly correlated with foliar EFN volume, but FN was weakly correlated with bracteal EFN volume (Chamberlain and Rudgers, 2012) questions the physiological linkage hypothesis since bracteal and floral nectaries are spatially closer than floral and extrafloral nectaries, but they are not strongly correlated. We suggest that lack of any trade-off could also indicate that FN and EFN may be phenotypically integrated as a functional module for mutualist attraction. Although formal analyses are required to test this hypothesis, we think it is a strong possibility since *T. velutina* leaves are phenotypically integrated modules in which leaf economics, defensive and morphological traits covary and are ecologically linked (Damián et al., 2018). Whatever the drivers of these positive correlations may be, available evidence suggests that plants may experience fewer investment trade-offs among different functional traits than previously assumed.

## Conclusions

To our knowledge, trade-offs between extrafloral and floral nectar traits have been studied in 41 species from two genera: 37 *Gossypium* species (Chamberlain and Rudgers, 2012), and four *Turnera* species, including this study (Dutton et al., 2016; Villamil, 2017, Figure 2). Negative correlations or evidence for trade-offs have been found in only two of these species: *T. ulmifolia* and *T. subulata* (Dutton et al., 2016), representing less than 5% of the species studied. Although many more studies are required to shed light on quantitative trends of floral and extrafloral investment in plants, trade-offs between floral and extrafloral seem infrequent. On the other hand, evidence of direct conflict with patrolling ants reducing pollinator visitation frequency and duration, inducing inspection behaviors and increasing foraging time has been widely reported (Rudgers and Gardener, 2004; Ness, 2006; Chamberlain and Rudgers, 2012; Malé et al., 2012, 2015; Assunção et al., 2014; Koptur et al., 2015; Martínez-Bauer et al., 2015). We suggest that these two issues are not isolated, and hypothesize that positive correlations between FN and EFN investment in ant-plants may be a plant strategy to compensate or lure pollinators to apparently risky flowers.

## Data availability statement

The raw data supporting the conclusions of this manuscript will be made available by the authors upon request.

## Author contributions

NV conceived the ideas, all authors designed the experiments. NV conducted fieldwork, processed, and analyzed the data. NV led the writing of this paper, with critical inputs from KB and GS.

### Conflict of interest statement

The authors declare that the research was conducted in the absence of any commercial or financial relationships that could be construed as a potential conflict of interest.
